# Magnetic Resonance Spectroscopy for Detection of 2-Hydroxyglutarate as a Biomarker for IDH Mutation in Gliomas

**DOI:** 10.3390/metabo7020029

**Published:** 2017-06-19

**Authors:** Thomas Leather, Michael D. Jenkinson, Kumar Das, Harish Poptani

**Affiliations:** 1 Centre for Pre-clinical Imaging, Department of Cellular and Molecular Physiology, University of Liverpool, Liverpool L69 3BX, UK; hltleath@student.liverpool.ac.uk; 2 Institute of Translational Medicine, University of Liverpool, Clinical Science Centre, Lower Lane, Liverpool L9 7LJ, UK; michael.jenkinson@liv.ac.uk; 3 Department of Neurosurgery, The Walton Centre NHS Foundation Trust, Lower Lane, Liverpool L9 7LJ, UK; 4 Department of Neuroradiology, The Walton Centre NHS Foundation Trust, Lower Lane, Liverpool L9 7LJ, UK; Kumar.Das@thewaltoncentre.nhs.uk

**Keywords:** isocitrate dehydrogenase, glioma, glioblastoma, 2-hydroxyglutarate, magnetic resonance spectroscopy

## Abstract

Mutations in the isocitrate dehydrogenase (IDH)1/2 genes are highly prevalent in gliomas and have been suggested to play an important role in the development and progression of the disease. Tumours harbouring these mutations exhibit a significant alteration in their metabolism resulting in the aberrant accumulation of the oncometabolite 2-hydroxygluarate (2-HG). As well as being suggested to play an important role in tumour progression, 2-HG may serve as a surrogate indicator of IDH status through non-invasive detection using magnetic resonance spectroscopy (MRS). In this review, we describe the recent efforts in developing MRS methods for detection and quantification of 2-HG in vivo and provide an assessment of the role of the 2-HG in gliomagenesis and patient prognosis.

## 1. Introduction

The field of neuro-oncology has undergone a significant paradigm shift following the identification of mutations in the isocitrate dehydrogenase (IDH) one and two genes in glioma and gliomagenesis. Such mutations have been suggested to play an important role in the development and progression of intracranial gliomas, often marking the first genetic transformation to occur, and imparting a range of downstream effects on a myriad of cellular processes [1]. Mutations in IDH1/2 genes have been identified in 70–80% of World Health Organization (WHO) grade II-III gliomas, and these mutations are particularly prevalent in grade IV secondary glioblastomas (GBM) arising as a progression from lower grade tumours, suggesting such mutations may act as a driving force behind malignant progression [2,3]. Mutations in IDH1/2 confer a gain-of-function neomorphic enzymatic activity, resulting in the aberrant production and subsequent accumulation of 2-hydroxygluarate (2-HG), which has been suggested to be an oncometabolite for this genetic mutation [4]. Magnetic resonance spectroscopy (MRS) has been identified as a tool in the diagnosis of IDH mutant gliomas via the non-invasive detection of 2-HG [5,6,7,8,9]. Alongside diagnostic applications, non-invasive detection and quantification of 2-HG levels within the mutant gliomas is highly desirable for the development of targeted treatment and response monitoring. The mutated pathway has been identified as a candidate target for novel therapies, and imaging strategies capable of accurately examining 2-HG levels will aid in longitudinal assessments of treatment response, whilst accelerating pharmaceutical translation from bench to bedside [10,11,12].

## 2. Biochemistry of IDH Mutation in Gliomas

**Figure 1:**
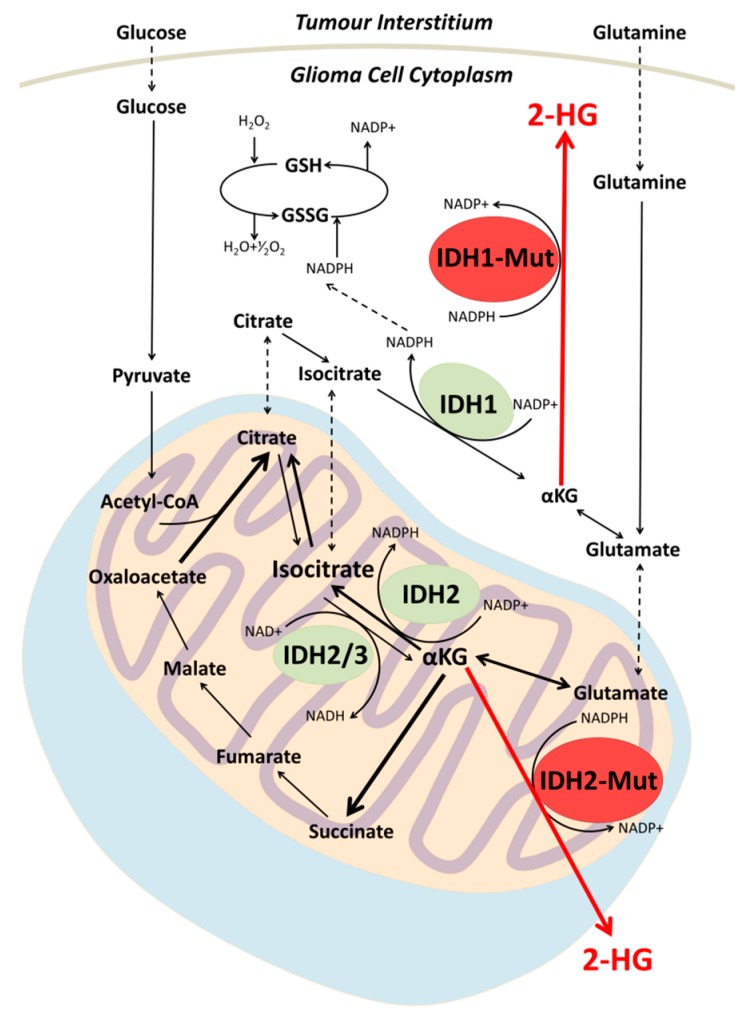
Simplified schematic of metabolic pathways associated with IDH1/2. Mutations in IDH1/2 catalyze the production of the oncometaboite 2-HG via reduction of αKG. Accumulation of 2-HG within the cytoplasm surpasses the detection threshold of magnetic resonance spectroscopy (MRS) at clinical field strengths, and therefore may be utilized as a biomarker for the non-invasive detection of the mutation. H_2_O_2_ = hydrogen peroxide, GSH = glutathione, GSSG = glutathione disulphide, H_2_O = water, NADP = nicotinamide adenine dinucleotide phosphate, NADPH = nicotinamide adenine dinucleotide phosphate hydrate, Acetyl-CoA = acetyl coenzyme A IDH1 = isocitrate dehydrogenase 1, IDH2 = Isocitrate dehydrogenase 2, 2-HG = 2-hydrozygluarate, αKG = alpha-ketoglutarate.

IDH is known to occur in three structural isoforms, namely IDH1, 2 and 3; however, only mutations in IDH1/2 have been identified in human gliomas [13]. IDH1 is localized to the cytoplasm and peroxisomes, whereas IDH2 is localised to the mitochondria [14]. Both of the IDH1/2 isoforms function to catalyse the oxidative decarboxylation of isocitrate into α-ketoglutarate (αKG) using NADP^+^ as a cofactor [15]. Mutations in both IDH1 and IDH2 are ubiquitously expressed within the tumour tissue; moreover, they consistently occur in a heterozygous manner and are most commonly observed in the IDH1 isoform. Remarkably, within gliomas, less than 90% of these point mutations occur at a single residue within the active site of the IDH1 isoform, where the Arginine 132 residue is replaced with a histidine (IDH1^R132H^) [1]. The much less frequent mutations in the IDH2 analog occur at Arginine 172, where the most common mutation is IDH2^R172K^. Alterations in the conformation of the catalytic site of the enzyme confer a gain-of-function neomorphic enzymatic activity, whereby mutant IDH1/2 catalyses the reduction of αKG into the oncometabolite 2-HG (Figure 1) [4]. The subsequent accumulation of the metabolite within mutated cells is facilitated by the heterozygous manner of the mutation and the dominant effect that the gain of function mutant imparts over the remaining wild type (WT) allele, which serves to provide the mutant enzyme with a continuous supply of αKG to be aberrantly reduced into 2-HG.

The full impact of accumulation of 2-HG within tumour cells is yet to be elucidated, but recent work suggests that it may have a pivotal role in altering the genetic, epigenetic and metabolic profile of the IDH mutant cells, driving the phenotype towards a more malignant state. One well-established mechanism of phenotypic alteration is inhibition of α-ketogluarate-dependant deoxygenases and histone demethylases, including the Ten Eleven Translocation (TET) family of 5-methlycytosine hydroxylases, leading to genome-wide alterations in methylation and histone patterns [13]. This proposed inhibition allows for IDH mutant gliomas to be classified into a distinct subgroup amongst intracranial tumours, whereby they exhibit a CpG island methylator phenotype and vastly altered histone methylation pattern [16]. This aberrantly methylated phenotype has been suggested to contribute to tumourgenesis. Interestingly, the occurrence of IDH mutations is highly restricted to a narrow spectrum, encompassing an exclusive list of malignancies. For example, these mutations are frequently observed in grades II to III gliomas and secondary GBM but not in primary GBM. The observed pattern in mutation occurrence suggests that its involvement in tumourgenesis may be linked to prevention of histone demethylation normally required for linage specific differentiation into terminally differentiated progenitors [17,18], in addition to altering the activity of chromosomal topological-regulating proteins [19]. A further molecular mechanism by which mutations in the IDH1/2 genes have been suggested to contribute to tumourgenesis is via the ability of accumulated 2-HG to stimulate activity of the egl-9 family hypoxia inducible factor (EGLN) prolyl 4-hydroxylases. Activation of these enzymes facilitates the ubiquitination and proteasomal degradation of hypoxia inducible factor, a process that has been shown to enhance the proliferation of astrocytes in culture [20]. 2-HG has also been suggested to indirectly contribute to increased exposure to oxidative stress, as the conferred neomorphic enzymatic activity observed in mutant cells incurs a reduction in NADPH levels, a molecule that normally functions as a prerequisite for the reduction of glutathione disulphide into the antioxidant glutathione (Figure 1) [21]. Although a decrease in glutathione levels increases exposure to free radicals and reactive oxygen species, it has been suggested that the cellular depletion of glutathione contributes to the increased sensitivity to adjuvant radiotherapy in IDH mutated gliomas [21].

One approach that has been adopted for probing IDH mutation uses ^13^C MRS to monitor the production of 2-HG from its substrate αKG in a rat glioma model [22]. This preclinical study used a Dynamic Nuclear Polarization labelled 1-^13^C αKG probe to examine the metabolic activity of tumour cells, providing information pertaining both the metabolic origin of the aberrantly produced 2-HG, as well as tracking of real-time metabolism. It was demonstrated that the injected hyperpolarized 1-^13^C αKG was converted to hyperpolarized 1-^13^C 2-HG in both cell lysates and in vivo in orthotopic tumours. In comparison to ^13^C, ^1^H MRS provides total, steady-state 2-HG levels present throughout the tumour environment, inclusive of both the intracellular and extracellular compartments. Alternatively, ^13^C MRS provides real-time, dynamic information on the metabolic fate of ^13^C α-KG, and thus directly probes the enzymatic activity of cytosolic mutant IDH1 in live glioma cells. The study of dynamic metabolite processes permitted by ^13^C MRS offers the opportunity to instantly gain a picture of the metabolic activity taking place within a tumour. The monitoring of active mutant IDH enzymes, as well as 2-HG synthesis, confirms the proposed biochemical pathway leading to the accumulation of 2-HG within glioma cells. In addition, utilization of a non-radioactive hyperpolarized 1-^13^C αKG probe highlights clinical applicability of such detection methods for the accurate tracking of oncogenic metabolic activity when monitoring disease progression; moreover, as IDH targeted therapies emerge, such techniques delivering high-quality information on 2-HG synthesis may be invaluable in the development and monitoring of novel compounds through clinical trials [23,24].

Taken together, the production and accumulation of 2-HG within IDH mutated glioma cells not only offers an interesting avenue for investigation into the underlying pathological events that contribute to glioma formation and progression, but correspondingly presents as a potential biomarker for diagnostic confirmation of IDH mutational status, in addition to prospective opportunities to monitor treatment and develop targeted therapies.

## 3. 2-HG as a Biomarker 

Although the downstream consequences of metabolic reprogramming observed in IDH mutated gliomas are yet to be fully elucidated, the incorporation of IDH status into the WHO diagnostic criteria indicate a significant diagnostic and prognostic role of the genetic abnormality. IDH status is currently confirmed by immunohistochemistry or genetic sequencing, requiring tissue samples obtained at surgery. There has been recent interest in developing non-invasive approaches that are able to utilise the aberrantly produced 2-HG as a biomarker for the mutation via detection through MRS.

As cancer treatments move towards personalised medicine, the need for robust and quantitative biomarkers becomes increasingly important. The ability of 2-HG to act as a surrogate marker of IDH status is highlighted by the fulfilment of several criteria. Firstly, it is known that > 99% of all IDH mutated cells exhibit 2-HG levels several orders of magnitude above the physiological trace amounts observed in healthy brain cells. In fact, accumulation of the metabolite occurs in such an extensive manner that its concentration reaches well into the millimolar (mM) range (5–35 mM) [25], far surpassing the sensitivity threshold of MRS at clinical field strengths (~1 mM) [26]. Secondly, there is virtually no background 2-HG present in healthy brain tissue. The 2-HG metabolite contains an asymmetric carbon within its backbone and is threrefore produced as two enantiomers, either D-*R*- or L-*S*- confirmation. The D-*R*-2-HG stereoisomer is the metabolite that is produced by the neomorph enzymes resulting from IDH mutation, whereas the L-*S*-2-HG isomer is produced as a result of limited oxygen availability within a tissue. Both steroisomers function to inhibt αKG-dependant enzymes. Physiologically, both enantiomers of this metabolite are rarely produced, and form only as transient errors of normal metabolism to be swiftly degraded by endogenous 2-HG dehydrogenases [4]. Finally, there is only one other known cause of elevated levels of 2-HG within brain tissue, a rare genetic disorder known as hydroxyglutaric aciduria, a condition recognised early in development due to the presentation of a range of additional clinical manifestations [27].

## 4. Prognostic Impact of IDH Mutation 

Overall survival (OS), treatment response and probability of malignant transformation are all factors that correlate with IDH mutation [24,28,29]. The prognosis of glioma is dependent on several factors including tumour grade as defined by the WHO classification [2]. Infiltrative low grade gliomas (LGGs) (grade II) have a median survival of 7–10 years, whilst high grade gliomas (HGGs) (WHO grade III and IV) have a poorer prognosis with a median OS ranging from a few months to a few years [29]. LGG (including oligodendroglioma, astrocytoma and mixed glioma) have an incidence rate of 0.63/100,000 adults per year [2] and hold the potential to undergo malignant transformation into WHO grade III anaplastic glioma or grade IV secondary GBM [30]. Mutations in IDH1/2 within LGGs are highly prevalent, and can be observed at a rate of 70–80% in all tumours within this subgroup. The influence of IDH mutation on LGG patients has been highlighted in the stratification of oligodendroglioma, astrocytoma and mixed glioma [31]. The study evaluated the accumulation of a number of additional genetic aberrations alongside IDH mutation, including hypermethylation of *O*-6-methylguanine DNA methyltransferase (MGMT) promotor region, 1p/19q co-deletion and TP53 mutation. It was reported that all genetic subsets, inclusive of IDH mutation, showed hazard ratios 3–5 times more likely to undergo malignant transformation [31]. Although LGG have been stratified with regards to the prognostic influence of IDH mutation, the importance of the genetic abnormality in this subset of tumours is still a topic of debate, as others have failed to identify such a significant correlation [32,33]. In addition to the increased risk reported by Leu et al. [31], a study conducted by Sanson et al. [34] observed a significant increase in OS in patients harbouring an IDH mutation. Conversely, other studies have not found a link between IDH status and OS [32,33,35]. Kim et al. reported no significant influence of IDH on progression free survival (PFS) or OS in a cohort of nearly 200 LGGs [33]. Furthermore, Juratil et al. failed to identify any predictive value of the mutation with regard to OS, PFS and mean time to malignant transformation (MTT) in a multivariate analysis [36]. This investigation did, however, identify that a higher rate of patients with mutated IDH were to develop into a secondary high grade glioma. A comprehensive analysis of cellular tricarboxylic acid cycle metabolites was conducted on a range of glioma and non-glioma patients, with both IDH mutant and WT genetic profiles. However, this study failed to identify 2-HG as an early biomarker in determining the prognostic course of glioma patients [35].

Whilst the influence of IDH mutation on the malignant progression of grade II gliomas is yet to be fully understood, the favourable impact on patient outcome for grade III gliomas is recognised. A number of studies have outlined the prognostic, rather than predictive influence of IDH mutation on grade III gliomas [32,33]. A prospective study by the European Organization for Research and Treatment of Cancer (EORTC) determining the prognostic significance of IDH mutation in anaplastic oligodendroglioma patients found that IDH mutant oligodendrogliomas had a better OS. However, the study did not find a link between IDH status and response to adjuvant radiation and chemotherapy [37]. Comprehensive, multiplatform integrative genomic analysis of low and intermediate grade gliomas demonstrated that lesions histologically identified as grade III display only a modest difference to those identified as grade II, whereby the majority of observed differences results in a slight increase in frequencies of chromosome 9p and 19q losses [38]. Such analyses highlight the suggested molecular progression through grade classification, initiated by mutation in IDH1/2 and acquisition of the CpG island methylator phenotype. Despite the modest differences in genetic profile, the confirmation of IDH mutation in grade III is considered to reflect improved PFS and OS [37].

In WHO grade IV GBM, IDH mutation has been repeatedly shown to be predictive of improved response to temozolomide treatment and radiotherapy in secondary GBM malignancies that have transformed from lower grade gliomas [39,40]. The presence of IDH mutation has additionally been associated with improved PFS and OS of secondary GBM [41,42,43]. Secondary GBM that also harbour hypermethylation of the MGMT promoter region alongside IDH1/2 mutation have the best prognosis [42]. Although the underlying molecular mechanisms behind IDH mutation and its proposed influences on diagnosis, prognosis and the suggested predictive capacity of the mutation are yet to be identified, there appears to be overwhelming evidence that this genetic alteration has a significant role to play in understanding the underlying pathology of gliomagenesis and tumour progression. There is therefore an unmet need for the development and implementation of a reliable and robust, non-invasive assay for the detection of this mutation.

## 5. Detection of 2-HG Via MRS

**Figure 2:**
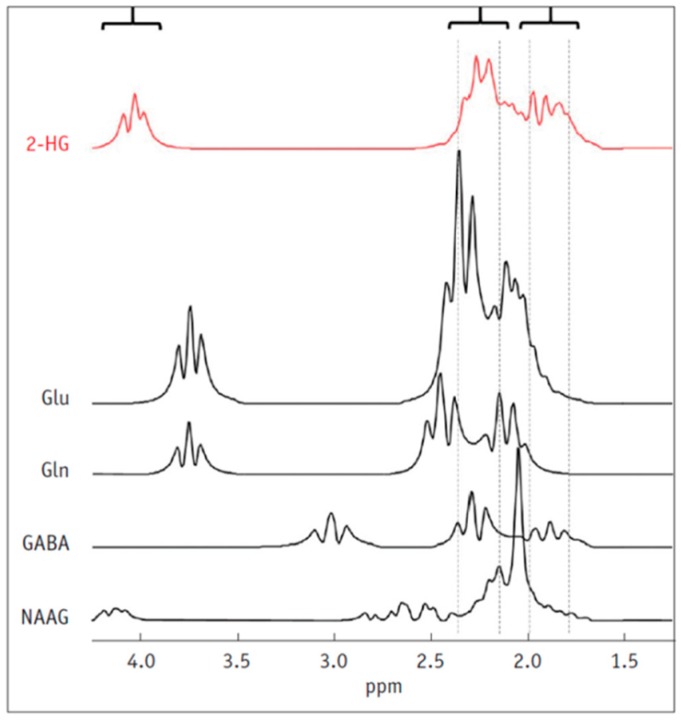
Simulated MR spectrum of 2-HG and overlapping background metabolites. The largest 2-HG peak at 2.5 ppm is heavily overlapped by a number of other metabolites found in abundance in brain tissue. 2-HG = 2-hydroxygluarate, Glu = Glutamate, Gln = Glutamine, GABA = gamma-aminobutyric acid, NAAG = *N*-acetlyaspartylglutamate. Taken with permission from Korean Journal of Radiology [48].

There are few known biomarkers that are as reflective of the underlying molecular changes seen in tumour pathology as 2-HG. In fact, accumulation of the metabolite is the only known direct consequence of a genetic mutation that can be detected and quantified using non-invasive imaging techniques. The markedly increased concentration of 2-HG in IDH mutant tumours has been demonstrated to correlate with tumour cellularity, and its accumulation may also be applied as a surgical adjunct to guide tissue sampling [5]. Although 2-HG represents as an attractive marker for diagnosis and monitoring of disease progression, unambiguous detection via MRS has proven difficult to establish. Complex spectral overlap by a number of metabolites, such as glutamate, glutamine and gamma-aminobutyric acid (GABA) (Figure 2), found in abundance within healthy brain tissue, often confound the identification and detection of 2-HG as well as compromise accurate quantification of metabolite concentration.

The 2-HG metabolite consists of five non-exchangeable scalar coupled protons comprising a complex 5-spin system (Figure 2) [44]. In addition to the 5-spin system, the J-coupling pattern of 2-HG leads to several multiplets with peaks identifiable at three spectral locations at a field strength of 3T centred around 4.02 ppm (H2), 2.25 ppm (H4 and H4′) and 1.9 ppm (H3 and H3′) [45]. The largest identifiable peak is located at 2.25 ppm due to the H4 and H4′ protons that are resonating proximally to each other [46]. However, there is a severe spectral overlap from a number of other physiologically occurring metabolites at this location, including glutamate (Glu), glutamine (Gln) and *N*-acetlyaspartylglutamate (NAAG), among others [5,6]. The influence of these overlapping resonances becomes more prevalent at clinically used field strengths, as lower fields and shorter acquisition times lead to inadequate spectral resolution, resulting in complex spectra that are challenging to resolve [45,47].

Several methods for detection of 2-HG in vivo have been proposed to optimise MRS for this application [5,6,7,8,49,50,51]. These encompass a range of acquisition and post-processing protocols designed to eliminate the confounding spectral overlap, and reliably quantify the 2-HG concentration in patients with IHD mutant glioma.

The severe overlap seen at the multiplets generated by the H4 and H3 peaks may induce a high rate of false positives when attempting to identify 2-HG if spectra are acquired via conventional ^1^H-MRS methods, and fitted using standard post-processing techniques. The feasibility of detection of 2-HG in vivo at clinical strengths, using a standard single-voxel double echo point-resolved spectroscopy (PRESS) sequence with a TE of 30 ms has been reported [8]. The acquired spectra were fitted with the LCModel software (http://s-provencher.com/lcmodel.shtml) using standard fitting algorithms [52]. Using this method, the authors reported a 26% false positive detection rate in IDH-WT tumours, due to severe overlap of the 2-HG signal with Glu and Gln at 2.25 ppm. Although short echo PRESS sequences are widely available in most clinical scanners, the high false positive detection rate limits the practical potential of this MRS method for detection of 2-HG. Ex vivo quantitation via liquid chromatography-mass spectrometry (LC-MS) did, however, show good correlation between MRS 2-HG measurements and true alterations in 2-HG concentration. Additionally, it was also identified that, although no changes in Glu or Gln could be observed in the IDH mutated subjects, there were additional metabolic alterations such as increased Choline (Cho). Cho levels were found to be further elevated in mutant tumours when compared to WT, a measurement validated via LC-MS, and a finding in line with previous studies that have examined metabolome-wide alterations seen in IDH mutated tumours [8,53]. Elevated Cho levels seen in IDH mutant tumours are suggested to reflect the increased cell density of mutant malignancies due to an IDH-mediated increase in cell proliferation [8]. Such observations obtained via MRS may be applied to furthering our understanding of the underlying mechanisms of the pathology, as elevated Cho levels have previously been correlated with tumour cell density, due to its involvement in cell membrane formation and proliferation [54,55]. Although the clinical capacity of the short TE PRESS acquisition method is limited by the high false positive detection rate, it may be applicable within a research setting, aimed at evaluating the role of IDH mutation in development and progression of glioma pathology.

**Figure 3:**
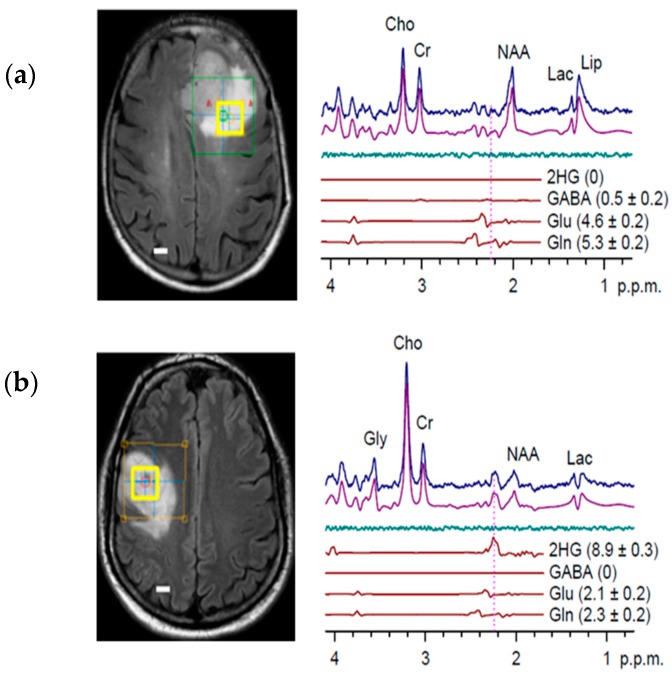
In vivo MR spectra of glioma patients acquired using single-voxel Echo Time (TE) 97 ms PRESS sequence at 3T, fitted using an LCModel. (**a**) grade IV Glioblastoma (GBM) IDH Wild Type (WT); (**b**) grade III Oligodendroglioma IDH1^R132H^. Taken with permission from Nature Medicine [2].

Improved spectral resolution and reduced overlap can be achieved when applying a longer TE to the standard PRESS sequences in order to take advantage of the difference in J-evolution seen in coupled spin systems [56]. The differential variation between coupled-spin system signal has been shown to allow adequate suppression of overlapping signal from background metabolites such as Glu and Gln, whilst maintaining sufficient signal-to-noise for accurate quantification of 2-HG (Figure 3) [57]. To take advantage of this method of spectral editing, an optimized PRESS sequence with a TE of 97 ms has been proposed [5]. Although spectral editing of uncoupled spins requires implementation of the longest possible TE to eliminate baseline noise, the complex 5-spin system of 2-HG requires optimization of interpulse durations and a specific TE in order to minimise signal loss from target metabolites whilst eliminating background noise [58]. To determine the optimal TE for isolation of the 2-HG signal, Choi et al. [5] conducted quantum mechanical simulations. It was concluded that a shorter first subecho time and longer second subecho yielded the greatest H4 resonances, cumulating to a TE of 97 ms (Figure 3) [5]. The study reported 100% specificity and sensitivity for the detection of 2-HG. Furthermore, various voxel sizes where tested to ascertain optimal parameters, whereby six out of the six voxels >8 mL in size belonging to WT tumours did not show any 2-HG signal [5]. The potential role of difference editing in MRS to untangle the 2-HG signal was also investigated. Difference editing, much like the long TE approach, takes advantage of J-coupling to generate spectral contrast [59]. This technique involves the application of frequency-selective radio-frequency pulses tuned at the H3 spins (1.9 ppm), the weak coupling partner to the H2 spins located at 4.0 ppm [5]. Alternate application of such pulses allows for selective 180° rotation of the H3 spins, producing a number of uneven H2 multiplets in the acquired subspectra that may then be subtracted from the overall signal, resulting in the cancellation of all other resonances unaffected by the 180° rotation [5]. In the study carried out by Choi et al. [5], difference editing was also applied to six subjects and yielded 100% sensitivity. However, employment of this method may not provide absolute metabolic profiles as it incurs imperatively long total TE, and, as such, may be subject to quantification errors that occur owing to signal loss due to relaxation [48]. Such errors associated with relaxation reduce the potential of the editing technique to track 2-HG for disease progression and appropriately monitor drug response.

In addition to the TE 97 ms PRESS sequence at 3T, the same group have recently proposed an optimized extended TE sequence at 7T [60]. The suggested method incurs a TE of 78 ms, taking into account the altered relaxation times at higher field strengths. The authors directly compared both the 3T and 7T methods, whereby five patients were scanned at both magnetic fields within the same week. It was apparent that application of the optimized PRESS sequence at higher field strengths conferred increased SNR, a point that was demonstrated by the reduction in mean 2-HG Cramer–Rao Lower Bounds, a measure of spectral fitting, from 8% at 3T to 5% at 7T [60]. Importantly, it was established that the mutual dependence of 2-HG and GABA signals were greatly reduced at 7T compared to 3T. The peak resonating at 2.25 ppm experiences a significant amount of spectral overlap from GABA resonances, and application of an extended TE PRESS sequence at 7T induced the opposite polarity and narrowing of signals from both 2-HG and GABA, allowing for enhanced signal separation when compared to the TE 97 ms PRESS at 3T [5,60].

To further establish the clinical applicability of the TE 97 ms PRESS sequence, de la Fuente et al. [61] demonstrated the limitations of the MRS sequence in detecting 2-HG, and the ability of MRS to non-invasively monitor response to cytoreductive treatments. They indicated that 2-HG-MRS increased sensitivity when applied to tumours with a volume >3.4 mL. In addition, they investigated the potential role of this sequence in monitoring response to glioma treatment, demonstrating that detected 2-HG levels correlated with tumour cellularity as measured by increased apparent diffusion coefficant and histopathology [61].

**Figure 4:**
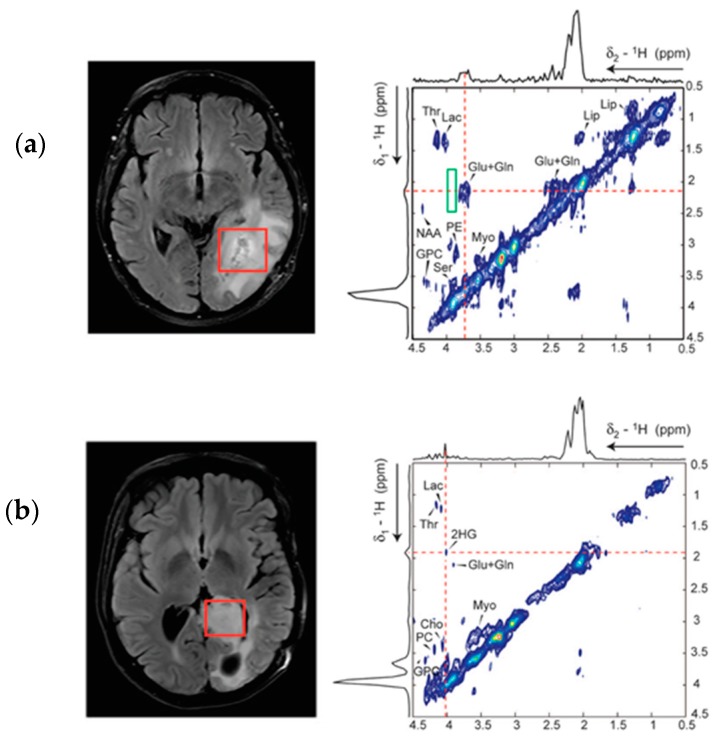
2D Correlation Spectroscopy (COSY) of human patients acquired at 3T, where the 3 × 3 × 3 cm^3^ voxel was placed over the tumour region, shown by the red box. (**a**) primary GBM patient IDH WT, there is a clear absence of cross peak in the region of 2-HG (demarked by green rectangle); (**b**) grade III anaplastic astrocytoma patient IDH1^R132H^; the 2-HG signal is unambiguously identifiable at the centre of crosshair intersection. Adapted with permission from Science Translational Medicine [5].

Unambiguous detection of 2-HG in glioma patients has been reported by using a 2D correlation spectroscopy (COSY) sequence at a field strength of 3T, to counteract the known cofounding spectral overlap observed in standard MRS spectra (Figure 4) [6]. 2D COSY allows for separation of the chemical shifts of spins that are scalar coupled to each other, and exploits the unlikely possibility that two metabolites would share identical chemical shifts in two dimensions. 2D COSY is particularly well suited for the application of identifying 2-HG, as the crosspeak generated by the metabolite appears in a spectral location shared by no other resonances. It was shown that 2D COSY was capable of unambiguous detection of 2-HG, providing full spectral information for accurate and reliable quantification. The abundance of spectroscopic information supplied by 2D methods represents a compromise between accuracy of measurements and complexity of acquisition. Although the most sensitive and specific detection method for identifying 2-HG, the clinical applicability of such a technique is limited by the extended acquisition time. This is an important factor when considering magnetic resonance imaging (MRI) scan time for patients, as current 2D COSY sequences are unlikely to be accommodated alongside clinical MRI brain tumour protocols within standard practice.

**Figure 5:**
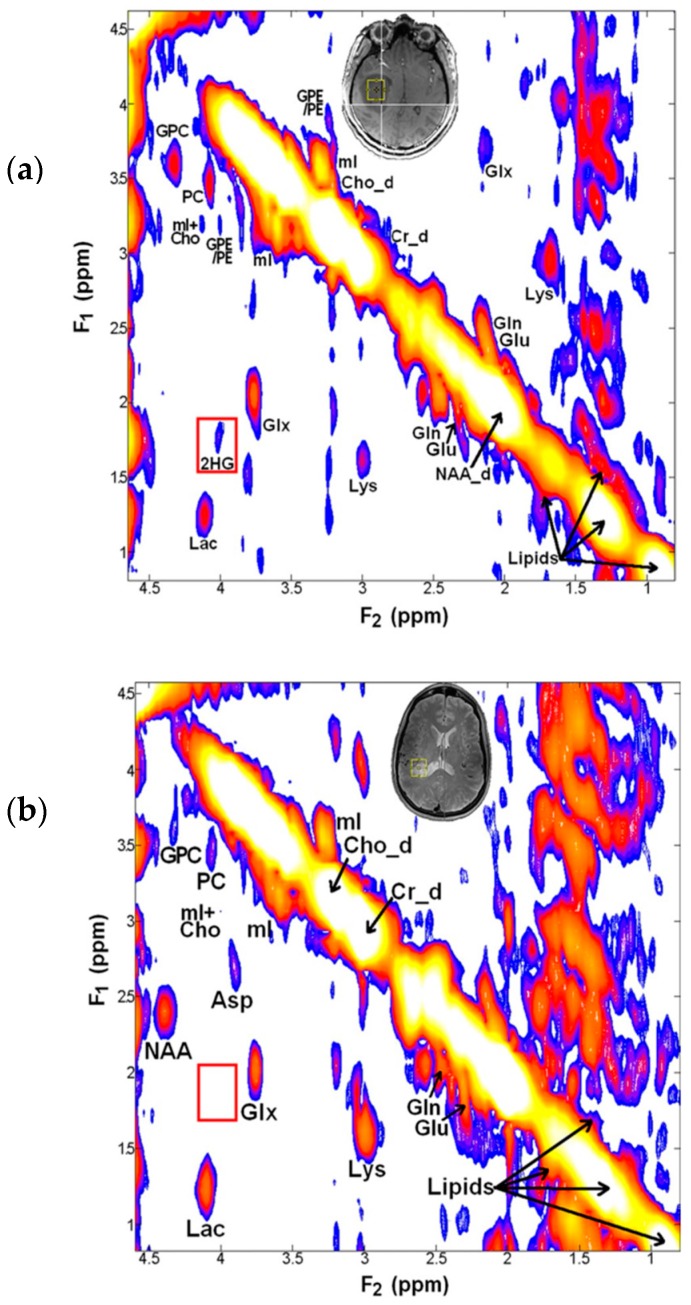
2D L-COSY of human glioma patients at 7T. (**a**) spectrum acquired from an 11.0 mL voxel from a IDH mutant grade III anaplastic astrocytoma patient; (**b**) spectrum acquired from an 8.8 mL voxel form a patient with a WT grade I ganglioglioma patient. The increased field strength not only benefitted the spectral separation of the 2-HG cross peak, but also allowed for increased spectral resolution and reliable quantification of other cancer associated metabolites such as Lactate. Taken with permission from Biomed Central [50].

2D L-COSY MRS at 7T has been used to not only identify the resonant peaks of 2-HG, but to further assess and resolve the spectra of other brain metabolites that may experience a change in concentration in the presence of IDH mutation (Figure 5) [50,62]. The 2D L-COSY method provided unambiguous detection of the metabolite in question; moreover, the higher field strength of 7T allowed for a reduction in voxel size and proportionally higher spectral separation, facilitating increased specificity of detection and quantification of 2-HG [50]. Whilst the clinical translational potential of the suggested 7T method is limited due to the high field strength, the 2D L-COSY proposed by Verma et al. may prove an excellent tool to further appreciate and investigate the shift in the metabolic profile of IDH mutated tumours. For example, the study demonstrated reliable quantification of increased levels of lactate, a useful tool in grading tumours, as it has been shown to reflect changes in glycolysis and tissue perfusion [63]. Reliable quantification of lactate concentration has previously proven problematic when applied to standard MRS methods, due to intense overlapping resonances from lipids often found in brain neoplasms that may be circumvented by the use of 2D L-COSY [50,64].

Although 2D MRS presents as an attractive method for accurate, reliable and sensitive detection and quantification of 2-HG, along with other glioma-associated metabolites, the clinical potential of this modality is limited when considering the incorporation of non-invasive metabolic profiling into diagnostic procedures. An alternative non-invasive method for detection of 2-HG utilizes an optimized semi-localization by an adiabatic selective refocusing (semi-LASER) sequence at 7T capable of providing quantitative measurements adequate enough to differentiate between cytosolic IDH1 mutant and mitochondrial IDH2 mutant [7]. Spectral changes induced by the presence of IDH mutation were characterised, and feature abnormalities were input into Fisher Linear Discriminant Analysis, where the resulting plot categorised two distinct cluster patterns, pertaining to IDH1 and IDH2 mutation. This detailed analysis was possible as a result of vastly improved localisation of the spectral volumes facilitated by employing a semi-LASER pulse sequence in combination with outer volume suppression, to eliminate contaminating signals from the area outside the volume of interest. The resultant spectra were free from artefacts normally generated from areas of surrounding tissue, and exhibited a flat baseline between the spectral locations of 1.6 ppm and 4.2 ppm [7].

**Figure 6:**
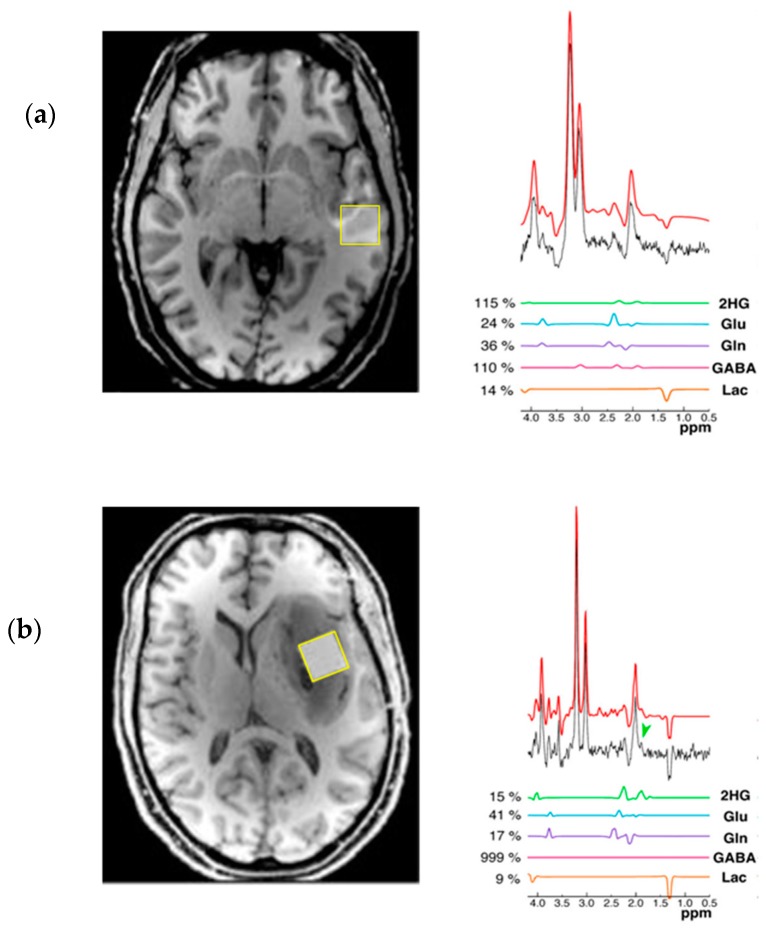
In vivo spectra acquired at 3T using an optimized semi-LASER sequence with a TE of 110 ms, the voxel was placed over area demarked by yellow box with LCModel fits and Cramer–Rao lower bounds (%) displayed for Glutamte, Glutamine, Gamma-anminobutyric acid and Lactate shown. (**a**) IDH WT glima patient, there is an unambiguous absence of 2-HG peak; (**b**) IDH1^R132H^ glioma patient where the 2-HG peak is easily identifiable. Adapted with permission from tomography [51].

In addition to the method for detection and quantification proposed at 7T, the same group has recently established a method for improved localisation of 2-HG detection at 3T by applying an optimized long-TE semi-LASER pulse sequence [51]. The use of broadband adiabatic localisation on strongly coupled spins allows for the acquisition of 2-HG spectra with a peak identifiable at 1.9 ppm that retains many features of the peak expected using ideal hard pules [51,65]. The outlined 3T semi-LASER method has been directly compared to the TE 97 ms PRESS sequence developed by Choi et al. [5]. The loss of the H3/H3′ peak as seen in TE 97 ms PRESS was circumvented by the use of adiabatic refocusing pulses in the semi-LASER sequence. Application of such refocusing pulses result in significantly reduced compartmental artefacts that would normally arise from extended J-evolution times under enhanced chemical shift displacements seen when narrow bandwidth RF pulses are applied [51,66]. The overall effect is total refocusing of the resonance at 1.9 ppm (H3 spins), which, when combined with diminished Glu and Gln peaks, allows for reliable detection of 2-HG (Figure 6). The significance of this method is highlighted by its translational potential and clinical applicability. Semi-LASER is a sequence that is increasingly being made available on 3T MRI within the clinical setting, and could be used for non-invasive in vivo assessment of IDH mutational status.

The mutated IDH pathway represents a possible target for novel glioma therapies, and, as such, there has begun a significant shift towards development of imaging strategies for the reliable longitudinal assessment and quantification of 2-HG levels during treatment and throughout clinical trials. Andronesi et al. propose a 3D functional spectroscopic mapping technique for the monitoring of 2-HG during clinical trials of drugs that may target the mutated pathway [49]. In order to quantify spatiotemporal changes of metabolite levels associated with IDH mutated glioma, a novel analysis metric was proposed, termed functional spectroscopic mapping (fSM) [49]. The resultant 3D analytical framework is able to provide comprehensive metabolic information over time. Standard single voxel or single slice MRS may often incur a sampling bias when probing for 2-HG in longitudinal analysis due to positional errors; however, 3D metabolite mapping is able to reduce the variance of spectral measurements obtained throughout the trial period [46].

In addition to the in vivo detection methods described above, efforts to characterize the metabolic profile of IDH1 mutated gliomas using ex vivo spectroscopic techniques have also been correlated with in vivo MRI parameters [9]. In a study conducted by Elkhaled et al. [9], image-guided biopsies were acquired and subsequently evaluated using magic angle spinning NMR spectroscopy. Although they failed to determine a link between tumour grade and relative abundance of 2-HG, a number of metaboites accosicated with tumour progression were correlated with the oncometabolite levels. Choline-containing species are some of the best spectroscopic markers for tumour evaluation and have been significantly utilized to determine tumour cellularity. In the study conducted by Elkhaled et al. [9], total choline was shown to significantly correlate with 2-HG levels, indicating the role of detection of the metabolite in determining phenotypic characterisation of glioma [9]. Moreover, the histoplathology of the tissue samples was assessed, where both immunostaining and histological investigations were used to assess for a number of parameters, including mitotic activity, relative tumour content and cellular density. 2-HG levels were found to correlate with a number of histopathological markers for mitotic activity, elevated tumour score and celluar density [9]. The strong correlations bewteen 2-HG and increased cellularity of IDH mutant tumours fall in line with the hypothesis that there is increased cellularity in tumours where 2-HG is present. These findings may also play an important role in the further development of in vivo MRS for both diagnosis and treatment response monitoring. If 2-HG levels can confidently aid in non-invasivly determining histopathological characteristics of glioma, the acceleration of novel treatments and the diagnostic accuracy of pre-surgical MRI could greatly benefit.

## 6. Treatment Advances for IDH Mutant Glioma

The emergence of IDH mutation and its role in glioma formation and progression has opened up the field of alteration of metabolism as a theraputic strategy once again. A selective inhibitor for IDHR^132H^ was identified [24] via high throughput screening, and it was demonstrated that, in a dose-dependent manner, the compound was able to abrogate the production of 2-HG. The inhibitor was shown to facilitate appropriate histone demethylation and re-expression of glial associated differentiation genes in endogenously mutated heterozygous astrocytoma cells [24]. Such compounds offer integral tools for the study of IDH mutations in glioma growth, where a further understanding can provide avenues for targeted therapies. Moreover, when patient derived glioma initiating cells were exposed to the DNA methyltransferase inhibitor Decitabine, it induced differentiation and restricted growth [10]. It was illustrated that administration of the non-cytotoxic, epigenetically targeted agent reversed DNA methylation marks associated with IDH mutation and promoted re-expression of genes associated with differentiation to control tumour growth [10]. With the emergence of targeted compounds, the need for a robust, reliable and clinically applicable method for probing IDH mutation in glioma becomes increasingly relevant. In addition to targeting the mutated pathway itself to manipulate the proposed oncogenic properties of mutant IDH and its products, recent studies have investigated the potential exploitation of the mutation in immunotherapeutic strategies. The rationale behind such an approach lies in IDH mutation acting as a tumour-specific neo-antigen, due to the ubiquitous expression and homogenous occurrence. The immunogenicity of an IDH1^R132^ vaccine was confirmed in mice by induction of CD4+ Major Histocompatibility Complex II-restricted mutation-specific antitumor immune response against cells expressing the IDH1^R132^ point mutation [11]. Other investigations exploring immunotherapeutic treatments have demonstrated the effectiveness in murine models, whereby administration of IDH1^R132^ specific vaccine caused a reduction in glioma growth rate [12].

## 7. Conclusions

In conclusion, methods capable of providing accurate quantification of 2-HG concentration for both research and diagnostic applications are set to have a significant impact on patient treatment paradigms. MRS may provide an invaluable tool to be applied in both the diagnosis and monitoring of IDH mutated glioma, in addition to allowing us to further our understanding of the genetic, epigenetic and molecular events that contribute to the pathology.

## References

[1] Waitkus M.S., Diplas B.H., Yan H. (2016). Isocitrate dehydrogenase mutations in gliomas. Neuro Oncol..

[2] Louis D.N., Perry A., Reifenberger G., von Deimling A., Figarella-Branger D., Cavenee W.K., Ohgaki H., Wiestler O.D., Kleihues P., Ellison D.W. (2016). The 2016 World Health Organization Classification of Tumors of the Central Nervous System: A summary. Acta Neuropathol..

[3] Yan H., Parsons D.W., Jin G., McLendon R., Rasheed B.A., Yuan W., Kos I., Batinic-Haberle I., Jones S., Riggins G.J. (2009). Mutations in Gliomas. N. Engl. J. Med..

[4] Dang L., White D.W., Gross S., Bennett B.D., Bittinger M.A., Driggers E.M., Fantin V.R., Jang H.G., Jin S., Keenan M.C. (2009). Cancer-associated IDH1 mutations produce 2-hydroxyglutarate. Nature.

[5] Choi C., Ganji S.K., DeBerardinis R.J., Hatanpaa K.J., Rakheja D., Kovacs Z., Yang X.L., Mashimo T., Raisanen J.M., Marin-Valencia I. (2012). 2-Hydroxyglutarate detection by magnetic resonance spectroscopy in IDH-mutated patients with gliomas. Nat. Med..

[6] Andronesi O.C., Kim G., Gerstner E., Batchelor T., Tzika A.A., Fantin V.R., Heiden M.G.V., Sorensen A.G. (2012). Detection of 2-Hydoxyglutarate in IDH-mutated Glioma Patients by Spectral-editing and 2D Correlation Magnetic Resonance Spectroscopy. Sci. Transl. Med..

[7] Emir U.E., Larkin S.J., De Pennington N., Voets N., Plaha P., Stacey R., Al-Qahtani K., McCullagh J., Schofield C.J., Clare S. (2016). Noninvasive quantification of 2-hydroxyglutarate in human gliomas with IDH1 and IDH2 mutations. Cancer Res..

[8] Pope W.B., Prins R.M., Thomas M.A., Nagarajan R., Yen K.E., Bittinger M.A., Salamon N., Chou A.P., Yong W.H., Soto H. (2012). Non-invasive detection of 2-hydroxyglutarate and other metabolites in IDH1 mutant glioma patients using magnetic resonance spectroscopy. J. Neurooncol..

[9] Elkhaled A., Jalbert L.E., Phillips J.J., Yoshihara H.A.I., Parvataneni R., Srinivasan R., Bourne G., Berger M.S., Chang S.M. (2012). Magnetic Resonance of 2-Hydroxyglutarate in Gliomas. Sci. Transl. Med..

[10] Turcan S., Fabius A.W., Borodovsky A., Pedraza A., Brennan C., Huse J., Viale A., Riggins G.J., Chan T.A., Turcan S. (2013). Efficient induction of differentiation and growth inhibition in IDH1 mutant glioma cells by the DNMT Inhibitor Decitabine. Oncotarget.

[11] Schumacher T., Bunse L., Pusch S., Sahm F., Wiestler B., Quandt J., Menn O., Osswald M., Oezen I., Ott M. (2014). A vaccine targeting mutant IDH1 induces antitumour immunity. Nature.

[12] Pellegatta S., Valletta L., Corbetta C., Patanè M., Zucca I., Riccardi Sirtori F., Bruzzone M., Fogliatto G., Isacchi A., Pollo B. (2015). Effective immuno-targeting of the IDH1 mutation R132H in a murine model of intracranial glioma. Acta Neuropathol. Commun..

[13] Yen K.E., Bittinger M.A., Su S.M., Fantin V.R. (2010). Cancer-associated IDH mutations: biomarker and therapeutic opportunities. Oncogene.

[14] McLendon R., Friedman A., Bigner D., Van Meir E.G., Brat D.J., Mastrogianakis G.M., Olson J.J., Mikkelsen T., Lehman N., Aldape K. (2008). Comprehensive genomic characterization defines human glioblastoma genes and core pathways. Nature.

[15] Parsons D.W., Jones S., Zhang X., Lin J.C., Leary R.J., Angenendt P., Mankoo P., Carter H., Siu I., Gallia G.L. (2008). An Integrated Genomic Analysis of human glioblastoma multiforme. Science.

[16] Noushmehr H., Weisenberger D.J., Diefes K., Phillips H.S., Pujara K., Berman B.P., Pan F., Pelloski C.E., Sulman E.P., Bhat K.P. (2010). Identification of a CpG Island Methylator Phenotype that Defines a Distinct Subgroup of Glioma. Cancer Cell.

[17] Lu C., Ward P., Kapoor G., Rohle D. (2012). IDH mutation impairs histone demethylation and results in a block to cell differentiation. Nature.

[18] Yang H., Ye D., Guan K.L., Xiong Y., Losman J.A., Kaelin W.G. (2013). IDH1 and IDH2 mutations in tumorigenesis: Mechanistic insights and clinical perspectives. Genes Dev..

[19] Flavahan W.A., Drier Y., Liau B.B., Gillespie S.M., Venteicher A.S., Stemmer-Rachamimov A.O., Suva M.L., Bernstein B.E. (2016). Insulator dysfunction and oncogene activation in IDH mutant gliomas. Nature.

[20] Koivunen P., Lee S., Duncan C.G., Lopez G., Lu G., Ramkissoon S., Losman J.A., Joensuu P., Bergmann U., Gross S. (2012). Transformation by the (R)-enantiomer of 2-hydroxyglutarate linked to EGLN activation. Nature.

[21] Molenaar R.J., Radivoyevitch T., Maciejewski J.P., van Noorden C.J.F., Bleeker F.E. (2014). The driver and passenger effects of isocitrate dehydrogenase 1 and 2 mutations in oncogenesis and survival prolongation. Biochim. Biophys. Acta Rev. Cancer.

[22] Chaumeil M.M., Larson P.E.Z., Yoshihara H.A.I., Danforth O.M., Vigneron D.B., Nelson S.J., Pieper R.O., Phillips J.J., Ronen S.M. (2013). Non-invasive in vivo assessment of IDH1 mutational status in glioma. Nat. Commun..

[23] Popovici-Muller J., Saunders J.O., Salituro F.G., Travins J.M., Yan S., Zhao F., Gross S., Dang L., Yen K.E., Yang H. (2012). Discovery of the first potent inhibitors of mutant IDH1 that lower tumor 2-HG in vivo. ACS Med. Chem. Lett..

[24] Bittinger M.A., Su S.M., Fantin V.R., Zhong C., Huang W., Ding J., Zhong C., Peng Y., Lai Z., Ding J. (2013). An Inhibitor of Mutant IDH1 Delays. Science.

[25] Viswanath P., Chaumeil M.M., Ronen S.M. (2016). Molecular Imaging of Metabolic Reprograming in Mutant IDH Cells. Front. Oncol..

[26] Serkova N.J., Brown M.S. (2012). Quantitative analysis in magnetic resonance spectroscopy: From metabolic profiling to in vivo biomarkers. Bioanalysis.

[27] Gokcen C., Isikay S., Yilmaz K. (2013). L-2 Hydroxyglutaric aciduria presenting with anxiety symptoms. BMJ Case Rep..

[28] Xia L., Wu B., Fu Z., Feng F., Qiao E., Li Q., Sun C., Ge M. (2015). Prognostic role of IDH mutations in gliomas: A meta-analysis of 55 observational studies. Oncotarget.

[29] Kleihues P., Ohgaki H. (1999). Primary and secondary glioblastomas: From concept to clinical diagnosis. Neuro Oncol..

[30] Ohgaki H., Kleihues P. (2005). Population-based studies on incidence, survival rates, and genetic alterations in astrocytic and oligodendroglial gliomas. J. Neuropathol. Exp. Neurol..

[31] Leu S., von Felten S., Frank S., Boulay J.-L., Mariani L. (2016). IDH mutation is associated with higher risk of malignant transformation in low-grade glioma. J. Neurooncol..

[32] Juratli T.A., Kirsch M., Geiger K., Klink B., Leipnitz E., Pinzer T., Soucek S., Schrok E., Schackert G., Krex D. (2012). The prognostic value of IDH mutations and MGMT promoter status in secondary high-grade gliomas. J. Neurooncol..

[33] Kim Y.H., Nobusawa S., Mittelbronn M., Paulus W., Brokinkel B., Keyvani K., Sure U., Wrede K., Nakazato Y., Tanaka Y. (2010). Molecular classification of low-grade diffuse gliomas. Am. J. Pathol..

[34] Sanson M., Marie Y., Paris S., Idbaih A., Laffaire J., Ducray F., Hallani S.E., Boisselier B., Mokhtari K., Hoang-Xuan K. (2009). Isocitrate dehydrogenase 1 codon 132 mutation is an important prognostic biomarker in gliomas. J. Clin. Oncol..

[35] Juratli T.A., Peitzsch M., Geiger K., Schackert G., Eisenhofer G., Krex D. (2013). A Biomarker for Malignant Progression. Neuro Oncol..

[36] Juratli T.A., Kirsch M., Robel K., Soucek S., Geiger K., Von Kummer R., Schackert G., Krex D. (2012). IDH mutations as an early and consistent marker in low-grade astrocytomas WHO grade II and their consecutive secondary high-grade gliomas. J. Neurooncol..

[37] Van Den Bent M.J., Dubbink H.J., Marie Y., Brandes A.A., Taphoorn M.J.B., Wesseling P., Frenay M., Tijssen C.C., Lacombe D., Idbaih A. (2010). IDH1 and IDH2 mutations are prognostic but not predictive for outcome in anaplastic oligodendroglial tumors: A report of the European Organization for Research and Treatment of Cancer Brain Tumor Group. Clin. Cancer Res..

[38] Gliomas L. (2015). Comprehensive, Integrative Genomic Analysis of Diffuse Lower-Grade Gliomas. N. Engl. J. Med..

[39] Combs S.E., Rieken S., Wick W., Abdollahi A., von Deimling A., Debus J., Hartmann C. (2011). Prognostic significance of IDH-1 and MGMT in patients with glioblastoma: One step forward, and one step back?. Radiat. Oncol..

[40] Millward C.P., Brodbelt A.R., Haylock B., Zakaria R., Baborie A., Crooks D., Husband D., Shenoy A., Wong H., Jenkinson M.D. (2016). The impact of MGMT methylation and IDH-1 mutation on long-term outcome for glioblastoma treated with chemoradiotherapy. Acta Neurochir..

[41] Chen J.R., Yao Y., Xu H.Z., Qin Z.Y. (2016). Isocitrate Dehydrogenase (IDH)1/2 Mutations as Prognostic Markers in Patients With Glioblastomas. Medicine.

[42] Yang P., Zhang W., Wang Y., Peng X.J., Chen B., Qiu X., Li G., Li S., Wu C., Yao K. (2015). IDH mutation and MGMT promoter methylation in glioblastoma: Results of a prospective registry. Oncotarget.

[43] Killela P.J., Pirozzi C.J., Healy P., Reitman Z.J., Lipp E., Rasheed B.A., Yang R., Diplas B.H., Wang Z., Greer P.K. (2014). Mutations in IDH1, IDH2, and in the TERT promoter define clinically distinct subgroups of adult malignant gliomas. Oncotarget.

[44] Bal D., Gryff-Keller A. (2002). 1H and 13C NMR study of 2-hydroxyglutaric acid and its lactone. Magn. Reson. Chem..

[45] Sener R.N.R. (2003). L-2 Hydroxyglutaric Aciduria: Proton Magnetic Resonance Spectroscopy and Diffusion Magnetic Resonance Imaging Findings. J. Comput. Assist. Tomogr..

[46] An Z., Ganji S.K., Tiwari V., Pinho M.C., Patel T., Barnett S., Pan E., Mickey B.E., Maher E.A., Choi C. (2016). Detection of 2-hydroxyglutarate in brain tumors by triple-refocusing MR spectroscopy at 3T in vivo. Magn. Reson. Med..

[47] Goffette S.M., Duprez T.P., Nassogne M.C.L., Vincent M.F.A., Jakobs C., Sindic C.J. (2006). L-2-Hydroxyglutaric aciduria: Clinical, genetic, and brain MRI characteristics in two adult sisters. Eur. J. Neurol..

[48] Kim H., Kim S., Lee H.H., Heo H. (2016). In Vivo Proton Magnetic Resonance Spectroscopy of 2-Hydroxyglutarate in Isocitrate Dehydrogenase-Mutated Gliomas: A Technical Review for Neuroradiologists. Korean J. Radiol..

[49] Andronesi O.C., Loebel F., Bogner W., Marjaska M., Heiden M.G.V., Iafrate A.J., Dietrich J., Batchelor T.T., Gerstner E.R., Kaelin W.G. (2016). Treatment response assessment in IDH-mutant glioma patients by noninvasive 3D functional spectroscopic mapping of 2-hydroxyglutarate. Clin. Cancer Res..

[50] Verma G., Mohan S., Nasrallah M.P., Brem S., Lee J.Y.K., Chawla S., Wang S., Nagarajan R., Thomas M.A., Poptani H. (2016). Non-invasive detection of 2-hydroxyglutarate in IDH-mutated gliomas using two-dimensional localized correlation spectroscopy (2D L-COSY) at 7 Tesla. J. Transl. Med..

[51] Berrington A., Voets N.L., Plaha P., Larkin S.J., Mccullagh J., Stacey R., Yildirim M., Schofield C.J., Jezzard P., Cadoux-Hudson T. (2016). Improved localisation for 2-hydroxyglutarate detection at 3T using long-TE semi-LASER. Tomography.

[52] Provencher S.W. (2001). Automatic quantitation of localized in vivo ^1^H spectra with LCModel. NMR Biomed..

[53] Reitman Z.J., Jin G., Karoly E.D., Spasojevic I., Yang J., Kinzler K.W. (2011). Profiling the effects of isocitrate dehydrogenase 1 and 2 mutations on the cellular metabolome. Proc. Natl. Acad. Sci. USA.

[54] Tedeschi G., Lundbom N., Raman R., Bonavita S., Jeff H.D., Jeffrey R.A., Giovanni D.C. (1997). Increased choline signal coinciding with malignant degeneration of cerebral gliomas: A serial proton magnetic resonance spectroscopy imaging study. J. Neurosurg..

[55] Dowling C., Bollen A.W., Noworolski S.M., McDermott M.W., Barbaro N.M., Day M.R., Henry R.G., Chang S.M., Dillon W.P., Nelson S.J. (2001). Preoperative proton MR spectroscopic imaging of brain tumors: Correlation with histopathologic analysis of resection specimens. Am. J. Neuroradiol..

[56] Allen P.S., Thompson R.B., Wilman A.H. (1997). Metabolite-specific NMR spectroscopy in vivo. NMR Biomed..

[57] Kim H., Thompson R.B., Hanstock C.C., Allen P.S. (2005). Variability of metabolite yield using STEAM or PRESS sequences in vivo at 3.0T, illustrated with myo-inositol. Magn. Reson. Med..

[58] Thompson R.B., Allen P.S. (1998). A new multiple quantum filter design procedure for use on strongly coupled spin systems foundin vivo: Its application to glutamate. Magn. Reson. Med..

[59] Mescher M., Merkle H., Kirsch J., Garwood M., Gruetter R. (1998). Simultaneous in vivo spectral editing and water suppression. NMR Biomed..

[60] Ganji S.K., An Z., Tiwari V., Mcneil S., Pinho M.C., Pan E., Mickey B.E., Maher E.A., Choi C. (2016). In vivo detection of 2-hydroxyglutarate in brain tumors by optimized point-resolved spectroscopy (PRESS) at 7T. Magn. Reson. Med..

[61] Izquierdo-Garcia J.L., Viswanath P., Eriksson P., Chaumeil M.M., Pieper R.O., Phillips J.J., Ronen S.M. (2015). Metabolic reprogramming in mutant IDH1 glioma cells. PLoS ONE.

[62] Sijens P.E., Levendag P.C., Vecht C.J., van Dijk P., Oudkerk M. (1996). ^1^H MR spectroscopy detection of lipids and lactate in metastatic brain tumors. NMR Biomed..

[63] De La Fuente M.I., Young R.J., Rubel J., Rosenblum M., Tisnado J., Briggs S., Arevalo-Perez J., Cross J.R., Campos C., Straley K. (2016). Integration of 2-hydroxyglutarate-proton magnetic resonance spectroscopy into clinical practice for disease monitoring in isocitrate dehydrogenase-mutant glioma. Neuro Oncol..

[64] Howe F.A., Barton S.J., Cudlip S.A., Stubbs M., Saunders D.E., Murphy M., Wilkins P., Opstad K.S., Doyle V.L., McLean M.A. (2003). Metabolic profiles of human brain tumors using quantitative in vivo ^1^H magnetic resonance spectroscopy. Magn. Reson. Med..

[65] Kaiser L.G., Young K., Matson G.B. (2008). Numerical simulations of localized high field ^1^H MR spectroscopy. J. Magn. Reson..

[66] Ramadan S., Mountford C.E. (2011). Adiabatic localized correlation spectroscopy (AL-COSY): Application in muscle and brain. J. Magn. Reson. Imaging.

